# Empagliflozin attenuates cardiac microvascular ischemia/reperfusion injury through improving mitochondrial homeostasis

**DOI:** 10.1186/s12933-022-01532-6

**Published:** 2022-06-15

**Authors:** Rongjun Zou, Wanting Shi, Junxiong Qiu, Na Zhou, Na Du, Hao Zhou, Xinxin Chen, Li Ma

**Affiliations:** 1 Heart Center, Guangdong Provincial Key Laboratory of Research in Structural Birth Defect Disease, Guangzhou Women and Children’s Medical Center, Guangzhou Medical University, Guangzhou, 510623 China; 2 Department of Paediatrics, Guangdong Provincial Key Laboratory of Research in Structural Birth Defect Disease, Guangzhou Women and Children’s Medical Center, Guangzhou Medical University, Guangzhou, 510623 China; 3 Child Healthcare Department, Guangdong Provincial Key Laboratory of Research in Structural Birth Defect Disease, Guangzhou Women and Children’s Medical Center, Guangzhou Medical University, Guangzhou, 510623 China; 4 Department of Cardiovascular Surgery, Sun Yat-sen Memorial Hospital, Sun Yat-sen University, 510120 Guangzhou, China; 5 Department of extracorporeal circulation, Guangdong Provincial Key Laboratory of Research in Structural Birth Defect Disease, Guangzhou Women and Children’s Medical Center, Guangzhou Medical University, 510623 Guangzhou, China; 6 Department of Nursing, Guangdong Provincial Key Laboratory of Research in Structural Birth Defect Disease, Guangzhou Women and Children’s Medical Center, Guangzhou Medical University, 510623 Guangzhou, China; 7 Senior Department of Cardiology, The Sixth Medical Center of People’s Liberation Army General Hospital, Beijing 100048 Beijing, China; 8 Department of Cardiology, Chinese PLA General Hospital, Medical School of Chinese PLA, 100037 Beijing, China

**Keywords:** Empagliflozin, Microvascular injury, Endothelial dysfunction, ischemia/reperfusion injury, Mitochondria

## Abstract

**Background:**

Empagliflozin has been reported to protect endothelial cell function, regardless of diabetes status. However, the role of empagliflozin in microvascular protection during myocardial ischemia reperfusion injury (I/R) has not been fully understood.

**Methods:**

Electron microscopy, western blots, immunofluorescence, qPCR, mutant plasmid transfection, co-immunoprecipitation were employed to explore whether empagliflozin could alleviate microvascular damage and endothelial injury during cardiac I/R injury.

**Results:**

In mice, empagliflozin attenuated I/R injury-induced microvascular occlusion and microthrombus formation. In human coronary artery endothelial cells, I/R injury led to adhesive factor upregulation, endothelial nitric oxide synthase inactivation, focal adhesion kinase downregulation, barrier dysfunction, cytoskeletal degradation and cellular apoptosis; however, empagliflozin treatment diminished these effects. Empagliflozin improved mitochondrial oxidative stress, mitochondrial respiration and adenosine triphosphate metabolism in I/R-treated human coronary artery endothelial cells by preventing the phosphorylation of dynamin-related protein 1 (Drp1) and mitochondrial fission 1 protein (Fis1), thus repressing mitochondrial fission. The protective effects of empagliflozin on mitochondrial homeostasis and endothelial function were abrogated by the re-introduction of phosphorylated Fis1, but not phosphorylated Drp1, suggesting that Fis1 dephosphorylation is the predominant mechanism whereby empagliflozin inhibits mitochondrial fission during I/R injury. Besides, I/R injury induced Fis1 phosphorylation primarily by activating the DNA-dependent protein kinase catalytic subunit (DNA-PKcs) pathway, while empagliflozin inactivated this pathway by exerting anti-oxidative effects.

**Conclusions:**

These results demonstrated that empagliflozin can protect the microvasculature by inhibiting the DNA-PKcs/Fis1/mitochondrial fission pathway during myocardial I/R injury.

## Background

Revascularization of occluded coronary arteries is a standard therapeutic approach for patients with myocardial infarction. However, revascularization also induces ischemia/reperfusion (I/R) injury in heart tissues, including cardiomyocytes and endothelial cells [1, 2]. I/R injury-induced endothelial dysfunction is associated with microvascular injury, which is characterized by perfusion defects, microvascular spasms, microthrombus formation and increased vascular permeability [3–5]. Cardiac microvascular I/R injury expands the infarction size and thus increases the peri-operative mortality of patients receiving revascularization therapy [6, 7]. Thus, despite the great success of therapies to reduce post-ischemic cardiomyocyte injury, additional molecular studies and therapeutic drugs are needed to address cardiac microvascular dysfunction during I/R injury.

Empagliflozin is an inhibitor of sodium/glucose cotransporter 2. Based on the findings of the EMPA-REG OUTCOME study, empagliflozin was originally used to treat type-2 diabetes in clinical practice [8]. However, based on the promising results of the EMPEROR-Reduced trial [9] and the EMPEROR-Preserved trial [10], the US Food and Drug Administration has also approved empagliflozin for the broader treatment of heart failure, regardless of diabetes status. Intensive research has been conducted to explain the cardioprotective potential of empagliflozin, and the anti-inflammatory [11], anti-oxidative [12], anti-apoptotic [13] and metabolic [14] effects of empagliflozin have been widely reported. These molecular mechanisms, in addition to hypoglycemic effects, may be the means by which empagliflozin enhances cardiomyocyte structure/function and thus suppresses the major adverse cardiovascular event rate in patients with heart failure [15, 16]. More importantly, several studies [17–19] have revealed that empagliflozin can protect endothelial cell function, regardless of diabetes status. The beneficial impact of empagliflozin on cardiac microvascular function has also been confirmed using non-invasive Doppler ultrasound imaging to assess the coronary flow velocity reserve [20]. Despite these exciting findings, the effects of empagliflozin on cardiac microvascular I/R injury are not fully understood.

Many studies have identified mitochondrial damage as a core pathogenic contributor to I/R injury-induced cardiac microvascular dysfunction [21–23]. Mitochondria-derived superoxides induce the oxidation of tetrahydrobiopterin to dihydrobiopterin, which prevents tetrahydrobiopterin from binding to endothelial nitric oxide synthase (eNOS), thus uncoupling eNOS and reducing nitric oxide production [24]. Moreover, when mitochondrial pro-apoptotic proteins such as cytochrome c and Smac infiltrate the cytosol, they activate caspase family members to promote apoptosis [25]. Mitochondria are also involved in endothelial mobilization, senescence, growth and proliferation [26–28].

It is now recognized that mitochondrial structural disorder precedes mitochondrial dysfunction [29, 30]. Increased mitochondrial fission reduces the mitochondrial membrane potential (MMP), augments mitochondrial reactive oxygen species (mtROS) production and activates mitochondria-dependent apoptotic pathways during cardiac microvascular I/R injury [30, 31]. Interestingly, our previous research and other studies have demonstrated that empagliflozin inhibits mitochondrial fission by suppressing the phosphorylation of dynamin-related protein 1 (Drp1) [32–34]. However, endothelial mitochondrial fission is induced by the phosphorylation of not only Drp1, but also mitochondrial fission 1 protein (Fis1) [35] and/or mitochondrial fission factor (Mff) [36]. It is poorly understood whether empagliflozin alters Fis1 or Mff phosphorylation in cardiac microvascular I/R injury.

Recently, we reported that Fis1 phosphorylation is an essential step in mitochondrial fission[37]. Oxidative stress promotes DNA damage [38, 39] and thus activates the DNA-dependent protein kinase catalytic subunit (DNA-PKcs), which binds to and phosphorylates Fis1. Phosphorylated Fis1 has an increased affinity for cytosolic Drp1, regardless of whether Drp1 is phosphorylated [35]. In light of the anti-oxidative activity of empagliflozin, we hypothesized that empagliflozin may prevent DNA-PKcs-induced Fis1 phosphorylation in endothelial cells, thus inhibiting mitochondrial fission, improving endothelial function and alleviating microvascular I/R injury.

## Materials and methods

### Murine myocardial I/R injury

 All experimental procedures that involved animals were approved by the University of Wyoming College of Health Sciences and Chinese PLA General Hospital, performed in accordance with the Animal Use Guidelines of Chinese PLA General Hospital, and consistent with the National Institutes of Health Guide for the Care and Use of Laboratory Animals (NIH publication No 85-23). 12-week-old C57BL/6J male mice were anesthetized with inhaled isoflurane (1.5–2%), intubated and ventilated with a small animal respirator (Harvard Apparatus). Then, a left thoracotomy was performed to expose the heart, and the left anterior descending coronary artery was ligated with an 8.0 surgical silk suture for 45 min to induce ischemia, followed by 2 h of reperfusion, as we previously reported [40]. Empagliflozin (10 mg/kg/d) was administered for seven days before myocardial I/R injury, in accordance with a previous study [32].

### Electron microscopy

Heart tissues (infarcted zone) were immerged in a fixative (2.5% glutaraldehyde) and stored overnight at 4 °C. The samples were then processed and analyzed as we previously described [41].

### Immunohistochemistry

Heart tissues (infarcted zone) were embedded in paraffin and dewaxed, as for hematoxylin and eosin staining. The sections were placed in a 3% H_2_O_2_ methanol solution at room temperature for 20 min to inactivate endogenous hydrogen peroxide. For the unmasking of antigens, the sections were microwaved for 15 min in 10 mmol/L citrate buffer, pH 6.0. Then, immunostaining was performed using the avidin: biotinylated enzyme complex method with an antibody against ICAM1 (1:500, Abcam, #179707). After being washed, the slices were incubated with an appropriate biotin-conjugated secondary antibody for one hour at room temperature, and then the color was developed using 3,3’-diaminobenzidine tetrahydrochloride as a chromogen. The sections were counterstained with Gill-2 hematoxylin (Thermo-Shandon, Pittsburgh, PA, USA). After being stained, the sections were dehydrated in increasing concentrations of ethanol and xylene. Immunohistochemical staining images were analyzed using Aperio ImageScope v12.32.8013 (Leica Biosystems).

### **HCAEC culture and sI/R injury*in vitro***

HCAECs (American Type Culture Collection, PCS-100-020™) were cultured with vascular basal cell medium containing 5 ng/mL vascular endothelial growth factor, 5 ng/mL epidermal growth factor, 5 ng/mL fibroblastic growth factor, 15 ng/mL insulin-like growth factor 1, 10 mM L-glutamine, 0.75 U/mL heparin sulfate, 1 µg/mL hydrocortisone, 50 µg/mL ascorbic acid, 1% amphotericin B, 1% penicillin-streptomycin and 10% fetal bovine serum. Hypoxia/reoxygenation was used to induce sI/R injury *in vitro.* In brief, the medium was changed to an ischemia-mimicking solution containing 5 mM 4-(2-hydroxyethyl)-1-piperazineethanesulfonic acid, 10 mM 2-deoxy-D-glucose, 139 mM NaCl, 12 mM KCl, 0.5 mM MgCl_2_, 1.3 mM CaCl_2_ and 20 mM lactic acid, pH 6.2, and then the cells were incubated under 100% nitrogen (O_2_ < 1%) at 37 °C for 45 min. The cultures were then returned to normal culture conditions (10% fetal bovine serum, 37 °C ambient air, 5% CO_2_) for 2 h. Empagliflozin (10 µM) was added to the HCAECs 12 h before the sI/R injury. For the induction of oxidative stress, HCAECs were treated with 0.3 mM hydrogen peroxide for six hours. NAC (10 mM) was added to the medium of HCAECs to reduce oxidative stress-induced DNA-PKcs activation in the presence of sI/R, based on a previous study [42].

### RNA extraction and quantitative polymerase chain reaction (PCR)

Total mRNA was isolated using a Total RNA Kit (Omega) and reverse transcribed using a Reverse Transcription Kit (Vazyme) according to the manufacturers’ instructions. Then, mRNA levels were determined using quantitative PCR with SYBR Green (Vazyme). The amount of each cDNA relative to the endogenous control (*β-actin*) was calculated using the 2^−ΔΔCt^ method. The following primer pairs were used: *TNFα* (Forward, 5’-AGATGGAGCAACCTAAGGTC-3’; Reverse, 5’-GCAGACCTCGCTGTTCTAGC-3’), *IL-6* (Forward, 5’-CAGACTCGCGCCTCTAAGGAGT-3’; Reverse, 5’-GATAGCCGATCCGTCGAA-3’), *MCP1* (Forward, 5’-GGATGGATTGCACAGCCATT-3’; Reverse, 5’-GCGCCGACTCAGAGGTGT-3’).

### Western blot analysis

Proteins from lysed cells were fractionated via sodium dodecyl sulfate polyacrylamide gel electrophoresis and transferred to nitrocellulose membranes. Nonspecific binding sites were blocked with 5% bovine serum albumin in Tris-buffered saline with Tween 20 (TBST: 120 mM Tris-HCl [pH 7.4], 150 mM NaCl, 0.05% Tween 20) for two hours at room temperature. The blots were incubated with specific primary antibodies (each diluted 1:1000) overnight at 4 °C. β-actin was used as a loading control. The membranes were then washed three times with TBST and incubated with horseradish peroxidase-conjugated secondary antibodies. Proteins were visualized using the Immobilon^™^ Western Chemiluminescent HRP substrate (Millipore, USA). The following antibodies were obtained from Abcam: ET-1 (#ab178454), eNOS (#ab199956), p-eNOS (#ab215717), Fak (#ab40794), Src (#ab133283), Drp1 (#ab184247), Fis1 (#ab156865), GAPDH (#ab8245), Tom20 (#ab186735), DNA-PKcs (#ab32566), F-actin (#ab130935) and Met (#ab51067). The following antibodies were obtained from Cell Signaling Technology: p-Drp1 (#4494), Mff (#86,668) and p-Mff (#49,281).

### Confocal analysis of the mitochondrial membrane potential (MMP), mitochondrial morphology and mPTP opening

The MMP was measured using an MMP assay kit (Solarbio, Beijing, China). Cells were stained with the unique fluorescence probe JC-1 at 37 °C for 20 min, and then were washed twice with phosphate-buffered saline. The fluorescence intensity of the cells was observed using a flow cytometer (BD FACSCalibur, NJ, USA) and a confocal laser scanning microscope (Olympus, Japan). Then, the average fluorescence intensity of green monomers and red aggregates was determined, and the ratio was calculated.

MitoTracker™ was used to detect changes in mitochondrial dynamics. For mitochondrial labeling, the cell samples were incubated with a 100 nM solution of MitoTracker™ Green FM (Thermo Fisher Scientific, MA, USA) for 30 min at 37 °C, 5% CO_2_. Images were obtained using a Nikon confocal microscope system and camera (Nikon Instruments, NY, USA). The fluorescent dye and length of mitochondria were measured using Image J software. We selected 10 random fluorescence fields from each group.

To track mPTP opening, we treated HCAECs with tetramethylrhodamine ethyl ester, based on our previous study. The fluorescent signal of tetramethylrhodamine ethyl ester was determined using a Nikon confocal microscope system and camera.

### Plasmids, lentiviruses, siRNA and reagents

Eukaryotic expression vectors encoding *Drp1*, *Fis1*, *DNA-PKcs* and *Met* were generated through the insertion of PCR-amplified fragments into a pcDNA3 vector (Invitrogen, CA, USA). Serine 616 was replaced with aspartic acid to establish the Drp1 phosphorylation-mimetic mutant (Drp1^S616D^). Threonine 34 was replaced with aspartic acid to construct the Fis1 phosphorylation-mimetic mutant (Fis1^T34D^). For lentiviral production, HEK293T cells were co-transfected with recombinant lentiviral vectors and a pPACK Packaging Plasmid Mix (System Biosciences) using MegaTran reagent (OriGene, Beijing, China). The target cells were infected with the lentiviruses according to the manufacturer’s instructions. siRNA against DNA-PKcs was established and transfected into cells according to our recent reports [37, 43].

### Immunoprecipitation assays

HCAECs were transfected with the indicated plasmids. After 24 h, the cells were lysed with ice-cold immunoprecipitation buffer (50 mM Tris-HCl, pH 8.0, 150 mM NaCl, 1 mM ethylenediaminetetraacetic acid, 1% Triton X-100 and 0.5% sodium deoxycholate) containing protease inhibitor cocktail tablets (04693132001, Roche), and the samples were centrifuged at 13,000x*g* for 15 min. Then, the cell lysates were incubated with Protein G-agarose beads (AA104307, Bestchrom) and the indicated antibodies overnight at 4 °C. After being washed with immunoprecipitation buffer, the immunocomplexes were collected and immunoblotted using the indicated primary antibodies and corresponding secondary antibodies. Clean-Blot IP Detection Reagent (21,230, Thermo Fisher Scientific) was used to prevent interference from denatured immunoprecipitation antibody fragments.

### Immunofluorescence staining, TUNEL assay and mitochondrial ROS detection

Samples were fixed with 4% paraformaldehyde and permeabilized with 0.1% saponin. The cells were then incubated with primary antibodies and the corresponding secondary antibodies. Images were acquired using a confocal microscope (LCS-SP8-STED, Leica). The TUNEL assay (Abcam, #ab66108) was conducted based on the manufacturer’s instructions. MtROS levels in HCAECs were measured using a Mitochondrial ROS Detection Assay Kit (Cayman, Cat#701,600) according to the manufacturer’s recommendations.

### ELISA

The activity levels of DNA-PKcs (DNA-PK Kinase Enzyme System, Cat. No. #V4106, Promega Corporation, USA), Met (MET Kinase Enzyme System, Cat. No. #V3361, Promega Corporation), caspase-3 (Human Cleaved Caspase-3 ELISA Kit, Abcam, #220,655), mitochondrial respiratory complex I (Complex I Enzyme Activity Microplate Assay Kit, Abcam, #ab109721) and mitochondrial respiratory complex II (Complex II Enzyme Activity Microplate Assay Kit, Abcam, #ab109908) were determined according to the manufacturers’ protocols for the respective kits. Intracellular ATP production was measured with an ATP Assay Kit (Abcam, #83,355) based on the manufacturer’s protocol. Serum Biochemical Analysis. Serum TnI (Elabscience, E-EL-M0086c), LDH activity (Lactate dehydrogenase (LDH) assay kit, Nanjing, jiancheng, China), and serum creatine kinase-MB (CK-MB) (creatine kinase MB isoenzyme Assay Kit, Nanjing jiancheng, China) were measured with an ATP Assay Kit (Abcam, #83,355) based on the manufacturer’s protocol.

### Statistical analyses

All data in this study were used to determine the means and standard deviations. Student’s t test was used to assess the differences between two groups. One-way analysis of variance was conducted to evaluate differences among three or more groups. Bonferroni analysis and Tamhane’s T2 analysis were performed as appropriate. P values < 0.05 were considered statistically significant.

## Results

### Empagliflozin reduces I/R injury-induced cardiac microvascular damage

**Fig. 1:**
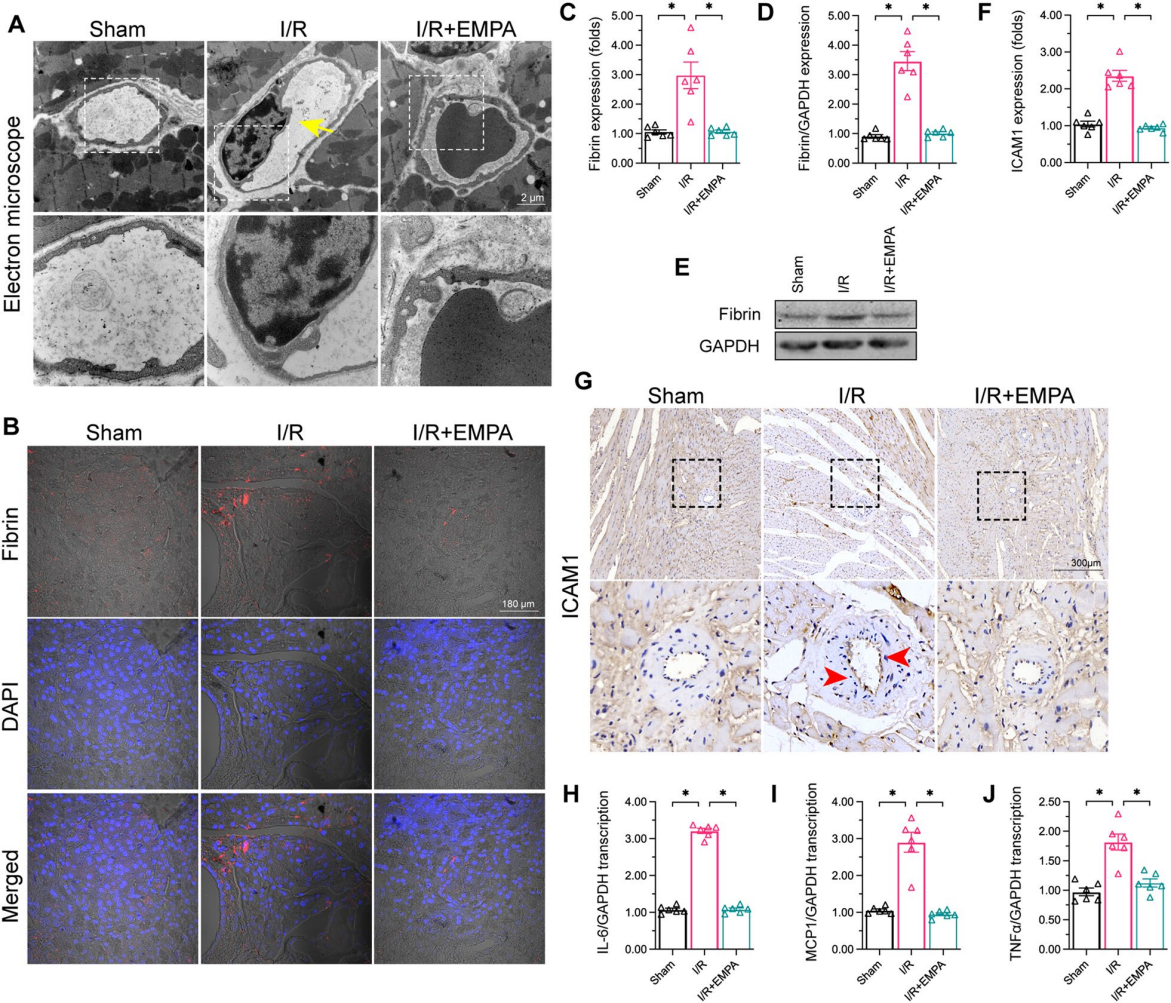
Empagliflozin reduces I/R injury-induced cardiac microvascular damage. Mice were subjected to 45 min of ischemia followed by two hours of reperfusion to induce cardiac I/R injury. Empagliflozin (EMPA, 10 mg/kg/d) was administered for seven days before myocardial I/R injury. **A** Electron microscopy was used to detect structural alterations in cardiac microvessels. Yellow arrows indicate the thickened wall and narrowed lumen. **B**, **C**. Immunofluorescence was used to detect fibrin accumulation in microvessels. **D**, **E** Proteins were isolated from reperfused heart and the expression of fibrin was determined by western blots. **F**, **G** Heart samples were collected after I/R injury for immunohistochemical analysis of ICAM1. **H** –**J** Total mRNA was isolated from reperfused hearts, and the mRNA levels of *IL-6*, *MCP-1* and *TNFα* were determined using quantitative PCR. Data are shown as mean ± SEM, n = 6 mice per group. *p < 0.05

To assess the effects of empagliflozin on cardiac microvascular damage during I/R injury, we treated mice with or without empagliflozin (10 mg/kg/d) for seven days before subjecting them to myocardial I/R injury or a sham operation. Then, we used electron microscopy to observe the microvascular structure. In mice exposed to I/R injury, the microvessel walls became thickened and narrowed; however, empagliflozin treatment reversed these alterations (Fig. 1A).

To determine whether the occluded microcirculation had induced microthrombus formation, we performed immunofluorescence analyses of fibrin in the cardiac microvessels. As expected, fibrin accumulation was greater in reperfused hearts than in sham-operated hearts (Fig. 1B, C). However, empagliflozin prevented the deposition of fibrin in the microcirculation, suggesting that this drug has anti-thrombotic effects (Fig. 1B, C). To validate these findings, we performed Western blotting, which confirmed that I/R injury promoted fibrin accumulation in heart tissues, whereas empagliflozin reversed this alteration (Fig. 1D, E).

In addition to luminal stenosis, the expression of adhesive factors such as intercellular adhesion molecule 1 (ICAM1) can increase the likelihood of microthrombus formation. As shown in Fig. 1F, G, I/R injury increased ICAM1 expression on the surface of microvessels, whereas empagliflozin supplementation reduced the abundance of ICAM1 in the microcirculation. Upregulated adhesive factors and increased microvessel permeability can stimulate the inflammatory response during I/R injury. Transcriptional analyses demonstrated that I/R injury augmented interleukin 6 (*IL-6*), tumor necrosis factor alpha (*TNFα*) and monocyte chemoattractant protein 1 (*MCP1*) mRNA levels, while empagliflozin treatment reduced the transcription of these inflammatory factors in heart tissues (Fig. 1H–J). Besides, the levels of cardiac injury markers (such as TnI, CK-MB and LDH) were also normalized by EMPA in the presence of I/R injury (Table 1). These results demonstrated that empagliflozin attenuates I/R injury-induced cardiac microvascular dysfunction.

### Empagliflozin sustains cardiac microvascular endothelial function after simulated I/R injury

**Fig. 2:**
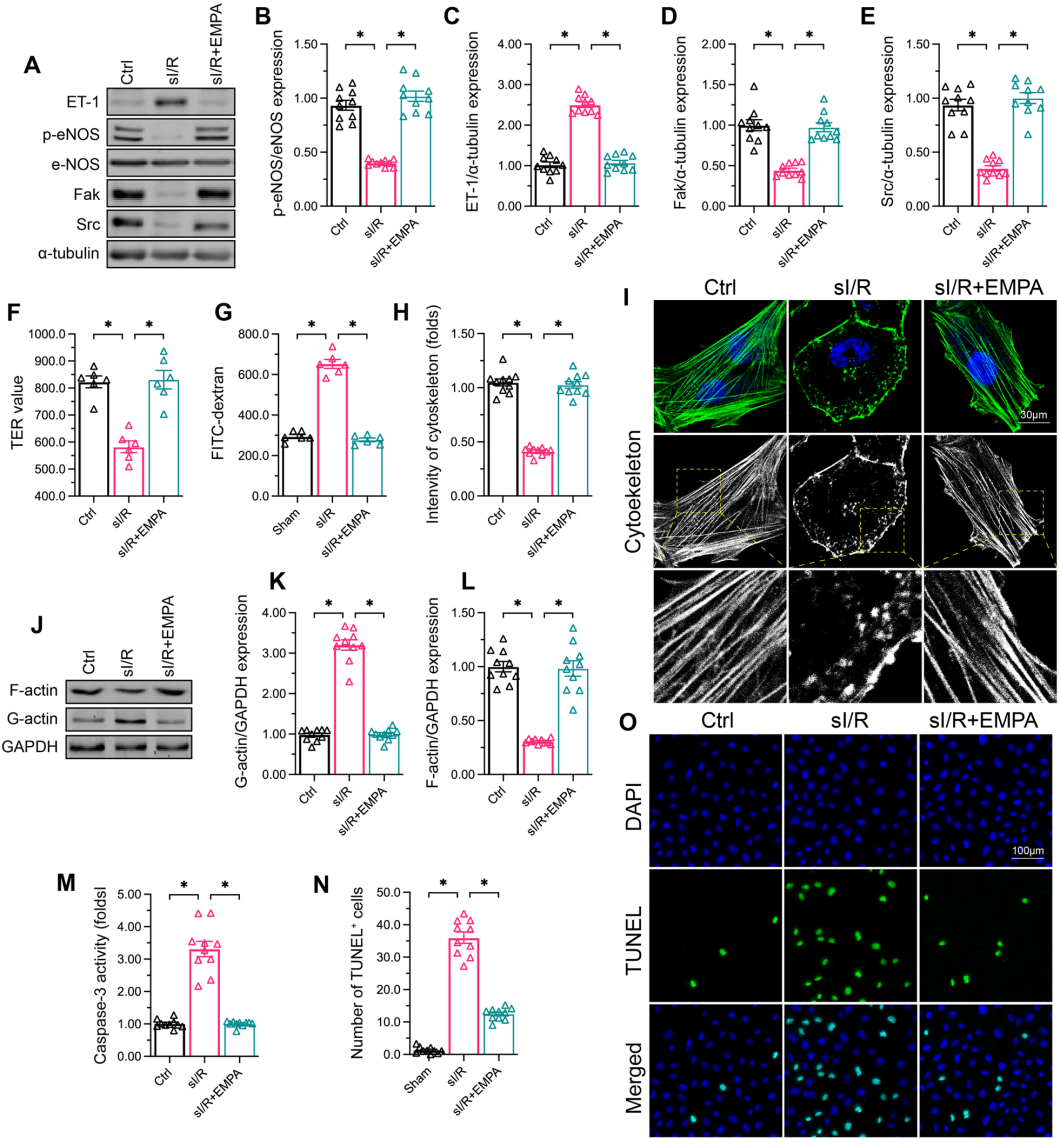
Empagliflozin sustains cardiac microvascular endothelial function after I/R injury. HCAECs were subjected to 45 min of hypoxia followed by two hours of reoxygenation to induce sI/R injury *in vitro.* The cells were incubated with empagliflozin (EMPA, 10 µM) for 12 h before sI/R injury. **A**–**E** Western blotting was used to assess the protein levels of ET-1, p-eNOS, Fak and Src in HCAECs following sI/R injury or empagliflozin treatment. **F**, **G** FITC-dextran clearance and TER assays were performed to determine the alterations of endothelial barrier function and integrity. **H**, **I** An immunofluorescence assay was used to observe cytoskeletal changes in HCAECs following sI/R injury. **J**–**L** Western blotting was used to determine F-actin protein levels in HCAECs following sI/R injury or empagliflozin treatment. **M** An ELISA was used to determine the activity of caspase-3, a marker of cell apoptosis. **N** TUNEL assay was used to observe the number of apoptotic endothelial cells in the presence of sI/R. Data are shown as mean ± SEM, n = ten independent cell isolations per group. *p < 0.05

Next, we evaluated the influence of empagliflozin on human coronary artery endothelial cells (HCAECs) following simulated I/R (sI/R) injury. Considering that the balance between endothelin 1 (ET-1) and eNOS fine-tunes endothelium-dependent vascular relaxation, we used Western blotting to examine the expression of these proteins in HCAECs. The results demonstrated that sI/R upregulated ET-1 expression and inhibited eNOS activity, whereas empagliflozin normalized the balance between these two proteins in sI/R-treated HCAECs (Fig. 2A–C). Src and focal adhesion kinase (Fak), the regulators of endothelial integrity and barrier function, were also downregulated in HCAECs upon sI/R injury, but were expressed at near-normal levels following empagliflozin treatment (Fig. 2A–E).

To further assess the effects of empagliflozin on barrier function and integrity, we performed fluorescein isothiocyanate (FITC)-dextran clearance and transendothelial electrical resistance (TER) assays. The remaining FITC-dextran content in HCAECs was significantly elevated following sI/R injury, indicating that endothelial hyperpermeability accelerated FITC-dextran accumulation (Fig. 2F–G). In addition, the TER value was reduced in sI/R-treated HCAECs, demonstrating that endothelial gap junctions were weakened. Empagliflozin administration not only reduced FITC-dextran deposition, but also enhanced the TER value (Fig. 2F–G), suggesting that empagliflozin can protect endothelial barrier function and integrity.

Previous research has indicated that cytoskeletal degradation, resulting from F-actin depolymerization into G-actin, is an early sign of endothelial dysfunction because it impairs cellular mobilization and stimulates apoptosis [44]. In HCAECs exposed to sI/R injury, the F-actin cytoskeleton became disorganized (Fig. 2H, I) and F-actin expression was reduced (Fig. 2J–L). On the other hand, a well-arranged F-actin cytoskeleton (Fig. 2H, I) and increased F-actin levels (Fig. 2J–L) were detected in empagliflozin-treated sI/R-injured HCAECs. The activity of the apoptosis marker caspase-3 increased upon sI/R injury, but decreased following empagliflozin treatment (Fig. 2M). Consistent with this observation, terminal deoxynucleotidyl transferase dUTP nick end labeling (TUNEL) analyses revealed that the number of apoptotic HCAECs increased upon sI/R stress and decreased in the presence of empagliflozin (Fig. 2N, O). These results demonstrated that empagliflozin sustains HCAEC function during sI/R injury.

### Empagliflozin restores mitochondrial homeostasis in endothelial cells

**Fig. 3:**
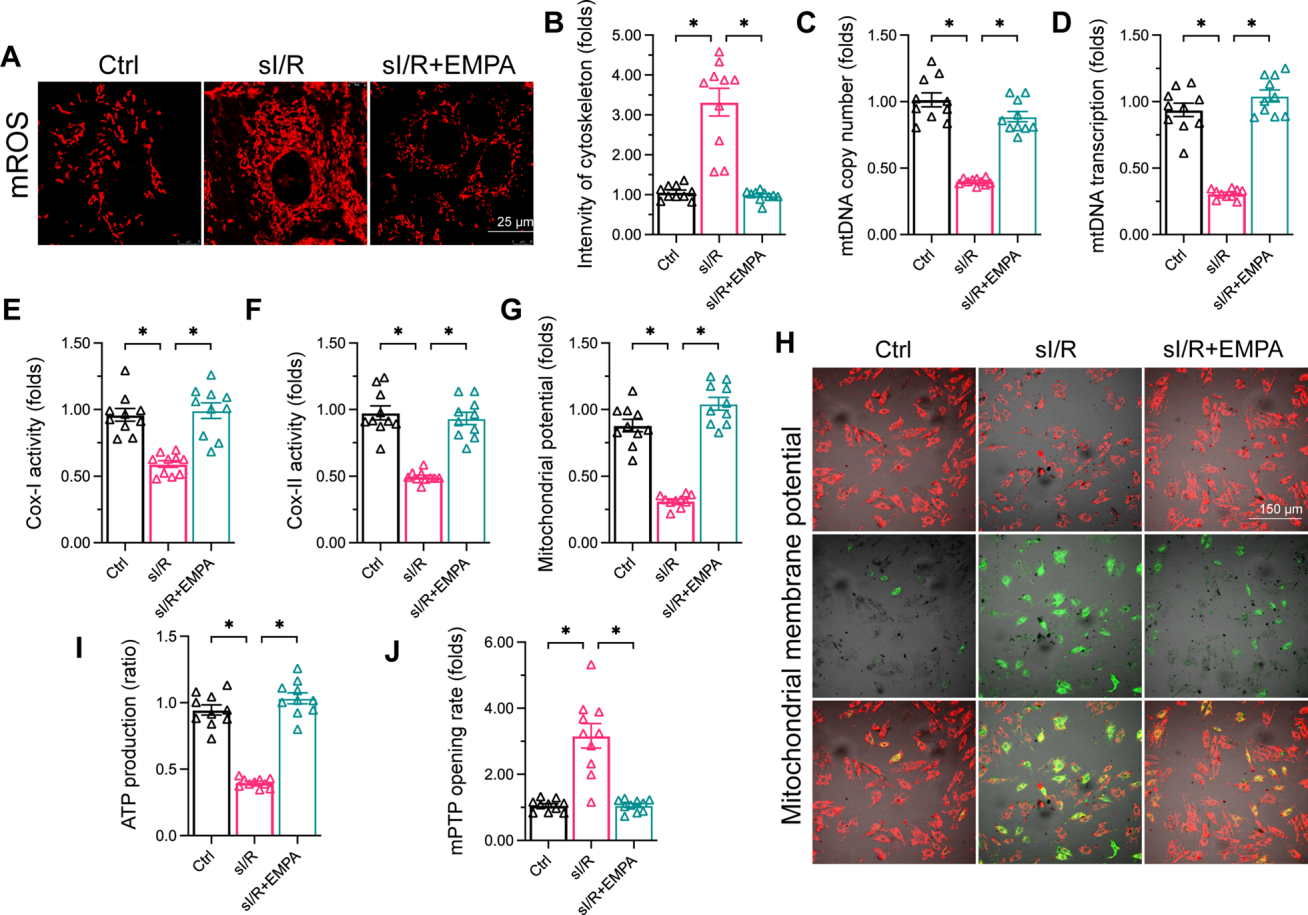
Empagliflozin restores mitochondrial homeostasis in endothelial cells. HCAECs were subjected to 45 min of hypoxia followed by two hours of reoxygenation to induce sI/R injury *in vitro.* The cells were incubated with empagliflozin (EMPA, 10 µM) for 12 h before sI/R injury. **A**, **B**. The mtROS levels in HCAECs were measured using a Mitochondrial ROS Detection Assay Kit. **C**, **D** qPCR was used to analyze the alterations of mitochondrial copy and transcription. **E**, **F** ELISAs were used to detect alterations in mitochondrial respiratory complexes I and II. **G**, **H** The MMP was measured using an MMP assay kit with the JC-10 fluorescence probe. **I** ATP production was measured with an ELISA. **J** The mPTP opening rate was determined using a tetramethylrhodamine ethyl ester fluorescence assay. Data are shown as mean ± SEM, n = ten independent cell isolations per group. *p < 0.05

**Fig. 4:**
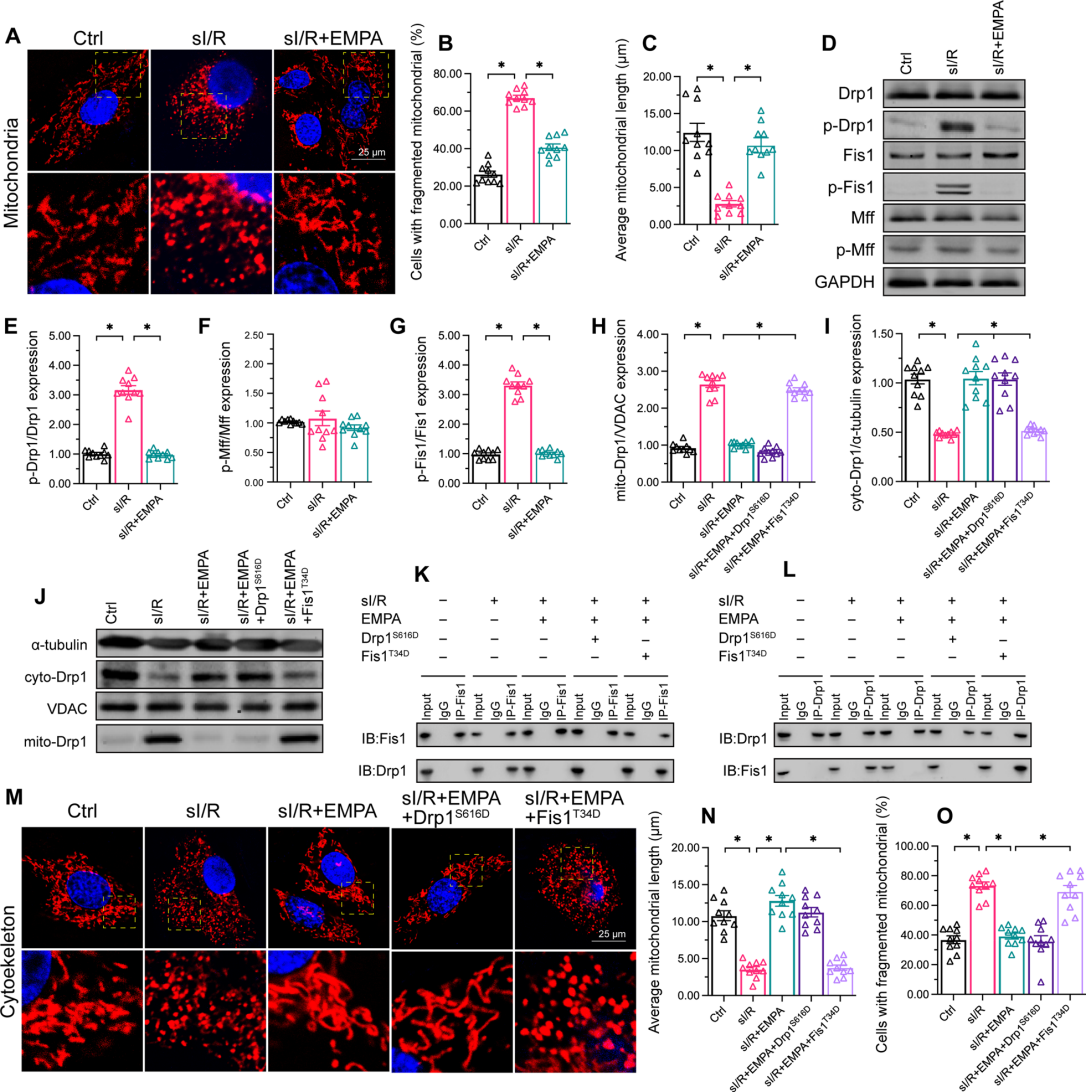
Empagliflozin suppresses mitochondrial fission by inhibiting Fis1 phosphorylation. HCAECs were subjected to 45 min of hypoxia followed by two hours of reoxygenation to induce sI/R injury *in vitro.* The cells were incubated with empagliflozin (EMPA, 10 µM) for 12 h before sI/R injury. **A**–**C** MitoTracker^™^ was used to detect changes in mitochondrial dynamics. The number cardiomyocyte with fragmented mitochondria as well as the average length of mitochondria were recorded. At least 100 mitochondria from 10 HCAECs were used to evaluate the number of HCAECs with fragmented mitochondria. **D**–**G** Western blotting was used to determine p-Drp1, p-Fis1 and p-Mff protein levels in HCAECs following sI/R injury or empagliflozin treatment. **H**–**J** Western blotting was used to assess cytoplasmic and mitochondrial Drp1 protein levels. HCAECs were transfected with a Drp1 phosphorylation-mimetic mutant (Drp1^S616D^) or a Fis1 phosphorylation-mimetic mutant (Fis1^T34D^) in the presence of empagliflozin. **K**–**L** The binding between Drp1 and Fis1 was determined using a co-immunoprecipitation assay in the presence of empagliflozin. HCAECs were transfected with Drp1^S616D^ or Fis1^T34D^. **M**–**O** Immunofluorescence staining was used to observe the mitochondrial morphology in HCAECs transfected with Drp1^S616D^ or Fis1^T34D^. Data are shown as mean ± SEM, n = ten independent cell isolations per group. *p < 0.05

Mitochondrial damage has been associated with endothelial dysfunction during sI/R injury [Figs. 3, 4]. Thus, we explored the effects of empagliflozin on endothelial mitochondrial function. We found that sI/R injury triggered mtROS production, whereas empagliflozin treatment neutralized excessive mtROS in HCAECs (Fig. 3A, B). Oxidative stress may damage the mitochondrial genome. As shown in Fig. 3 C, D, sI/R injury reduced the mitochondrial DNA copy number and transcription in HCAECs, while empagliflozin treatment attenuated these effects. Since the mitochondrial respiratory complexes are partly encoded by mitochondrial DNA, we measured mitochondrial respiratory complex I and II activity levels. The activities of these two complexes in HCAECs were reduced upon sI/R injury, but were sustained following empagliflozin supplementation (Fig. 3E, F).

The mitochondrial membrane potential (MMP) is primarily determined by electron transmission through the mitochondrial respiratory complexes. We found that sI/R injury depolarized the MMP, whereas empagliflozin stabilized the MMP in sI/R-treated HCAECs (Fig. 3G, H). Since the MMP drives adenosine triphosphate (ATP) synthesis, we also assessed ATP production. ATP levels were reduced in sI/R-treated HCAECs; however, empagliflozin supplementation maintained the normal energy output following sI/R injury (Fig. 3I). Moreover, ATP reduction is also a result of mitochondrial membrane hyperpermeability. We found that sI/R promoted, whereas empagliflozin inhibited, the opening of mitochondrial permeability transition pore (mPTP) (Fig. 3J) in HCAECs.These results revealed that empagliflozin preserves mitochondrial function in HCAECs under sI/R stress.

### Empagliflozin suppresses mitochondrial fission by inhibiting Fis1 phosphorylation

Our previous studies identified mitochondrial fission as an initial signal of mitochondrial dysfunction [45–47]. Thus, we wondered whether empagliflozin could maintain mitochondrial homeostasis by inhibiting mitochondrial fission in HCAECs. Immunofluorescence analyses of mitochondrial structure revealed that HCAECs exposed to sI/R injury contained fragmented and round mitochondria (Fig. 4A–C). Empagliflozin treatment elongated the mitochondria and maintained the highly-connected mitochondrial networks in HCAECs (Fig. 4A–C). At the molecular level, mitochondrial fission is primarily induced by Drp1 and its receptors, including Mff and Fis1 [48]. Western blotting demonstrated that sI/R injury induced Drp1 phosphorylation and Fis1 phosphorylation, but had no influence on Mff phosphorylation (Fig. 4D–G). Empagliflozin treatment inhibited the phosphorylation of not only Drp1, but also Fis1 (Fig. 4D–G).

The phosphorylation of Drp1 accelerates its translocation from the cytoplasm to the surface of the mitochondria [49]. On the other hand, the phosphorylation of Fis1 increases its affinity for cytoplasmic Drp1 [35]. To determine whether empagliflozin repressed mitochondrial fission through one or both of these mechanisms, we used a phosphorylation-mimetic Drp1 mutant (Drp1^S616D^, in which serine 616 was replaced with aspartic acid to mimic p-Drp1^Ser616^) and a phosphorylation-mimetic Fis1 mutant (Fis1^T34D^, in which threonine 34 was replaced with aspartic acid to mimic p-Fis1^Thr34^). We transfected HCAECs with these mutant constructs in the presence of empagliflozin, and then evaluated Drp1 translocation (from the cytoplasm to mitochondria) and mitochondrial fission following sI/R injury.

Of note, Fis1^T34D^ transfection enhanced mitochondrial Drp1 (mito-Drp1) expression in HCAECs, despite the treatment of these cells with empagliflozin; however, Drp1^S616D^ transfection did not have this effect (Fig. 4H–J). Furthermore, a co-immunoprecipitation assay revealed that empagliflozin impaired the binding between Drp1 and Fis1 in sI/R-injured HCAECs, and this effect was abolished by Fis1^T34D^ rather than by Drp1^S616D^ transfection (Fig. 4K–L). A morphological immunofluorescence assay of mitochondria indicated that empagliflozin mostly repressed mitochondrial fission in sI/R-treated HCAECs. Transfection of HCAECs with Fis1^T34D^ prevented empagliflozin from inhibiting mitochondrial fission; however, transfection with Drp1^S616D^ did not have this effect (Fig. 4M–O). These findings suggested that empagliflozin suppresses mitochondrial fission under sI/R injury primarily by promoting Fis1 dephosphorylation, although empagliflozin can also induce Drp1 dephosphorylation.

### Fis1^T34D^ mutant transfection prevents empagliflozin from protecting HCAEC mitochondria

**Fig. 5:**
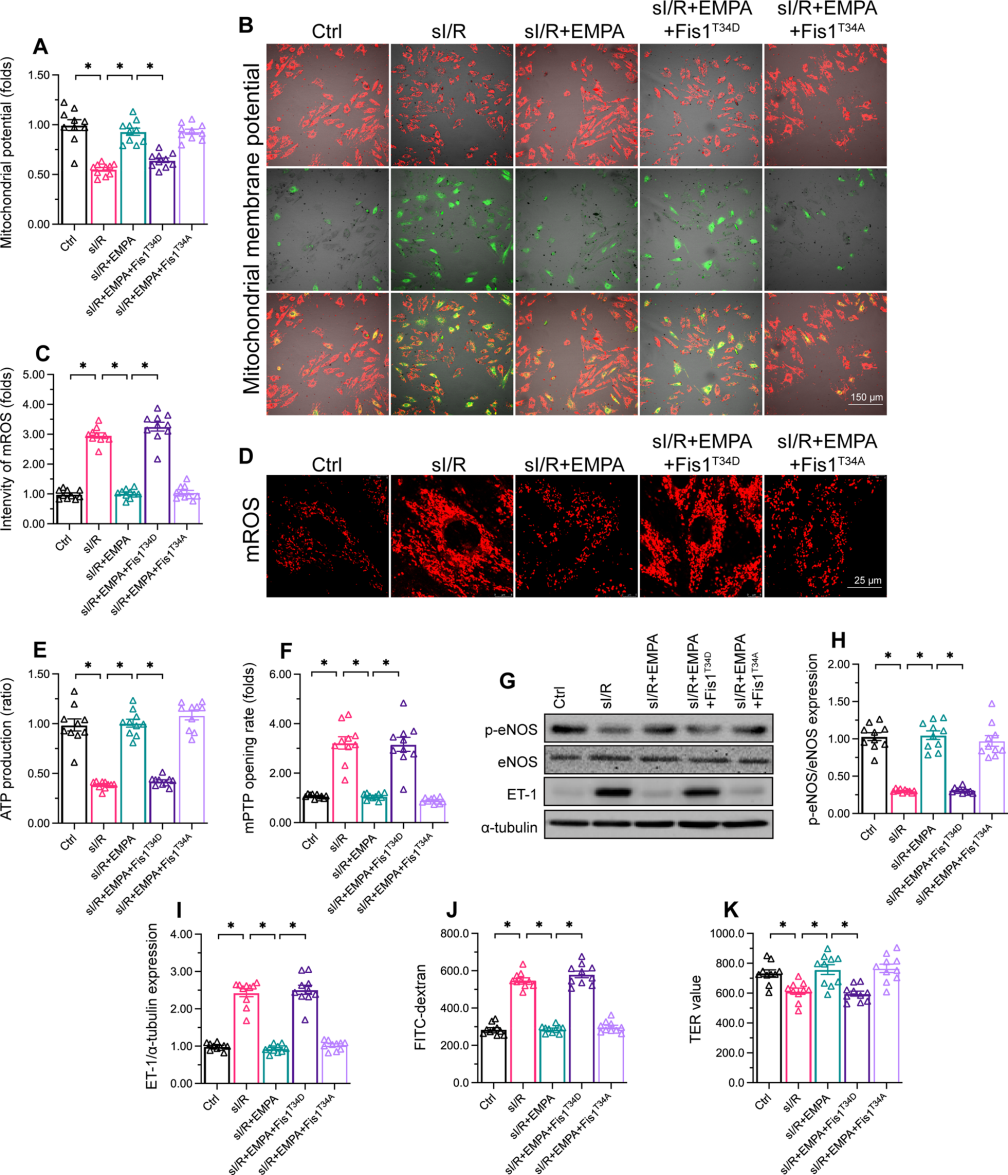
Fis1 mutant transfection prevents empagliflozin from protecting HCAEC mitochondria. HCAECs were subjected to 45 min of hypoxia followed by two hours of reoxygenation to induce sI/R injury *in vitro.* The cells were incubated with empagliflozin (EMPA, 10 µM) for 12 h before sI/R injury. HCAECs were transfected with a Fis1 phosphorylation-mimetic mutant (Fis1^T34D^) to activate mitochondrial fission in empagliflozin-treated sI/R-injured HCAECs, or were transfected with a Fis1 phosphorylation-defective mutant (Fis1^T34A^) to inhibit mitochondrial fission in sI/R-treated HCAECs. **A**,**B** The MMP was measured using an MMP assay kit with the JC-10 fluorescence probe. **C**,**D** The mtROS levels in HCAECs were measured using a Mitochondrial ROS Detection Assay Kit. **E** ATP production was measured with an ELISA. **F.** The mPTP opening rate was determined with a tetramethylrhodamine ethyl ester fluorescence assay. **G**–**I** Western blotting was used to assess ET-1 and p-eNOS protein levels in HCAECs following sI/R injury or empagliflozin treatment. **J**,**K** FITC-dextran clearance and TER assays were performed to determine the alterations of endothelial barrier function and integrity. Data are shown as mean ± SEM, n = ten independent cell isolations per group. *p < 0.05

To confirm that Fis1 dephosphorylation was the main mechanism whereby empagliflozin protected HCAECs and their mitochondria, we either transfected empagliflozin-treated HCAECs with the Fis1^T34D^ mutant, or transfected sI/R-injured HCAECs with a Fis1 phosphorylation-defective mutant (Fis1^T34A^, in which threonine 34 was replaced with alanine, as the negative control group). Then, we evaluated mitochondrial homeostasis and endothelial function. As shown in Fig. 5A, B, empagliflozin administration or Fis1^T34A^ transfection had similar effects in sustaining the MMP of sI/R-injured HCAECs. However, empagliflozin failed to maintain the MMP of sI/R-injured HCAECs transfected with Fis1^T34D^ (Fig. 5A, B). Empagliflozin supplementation also inhibited mtROS generation (Fig. 5C, D) and enhanced ATP production (Fig. 5E) in sI/R-injured HCAECs, while Fis1^T34D^ transfection abrogated these effects. Moreover, empagliflozin suppressed the mitochondrial permeability transition pore (mPTP) opening rate after sI/R injury in normal HCAECs, but not in HCAECs transfected with Fis1^T34D^ (Fig. 5F).

Western blotting demonstrated that empagliflozin treatment downregulated ET-1 but upregulated eNOS activity in sI/R-injured HCAECs; however, these alterations were undetectable in cells transfected with Fis1^T34D^ (Fig. 5G–I). Additionally, FITC-dextran clearance and TER assays confirmed that empagliflozin protected endothelial barrier function and integrity in HCAECs subjected to sI/R injury, whereas Fis1^T34D^ transfection nullified these beneficial actions (Fig. 5J and K). These data suggested that empagliflozin maintains mitochondrial homeostasis and endothelial function by suppressing Fis1 phosphorylation.

### Empagliflozin inhibits oxidative stress-induced DNA-PKcs activation

**Fig. 6:**
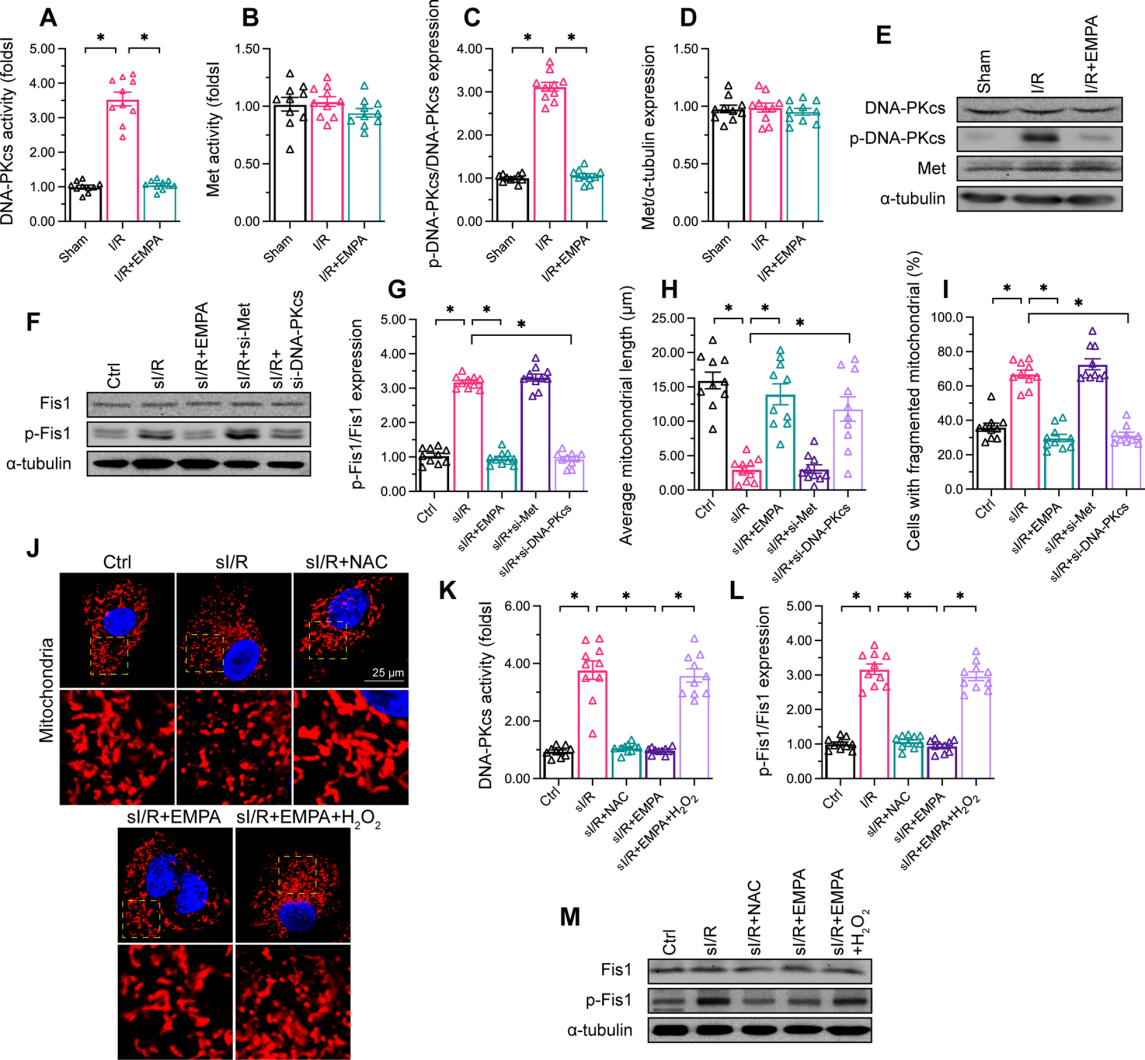
Empagliflozin inhibits oxidative stress-induced DNA-PKcs activation. HCAECs were subjected to 45 min of hypoxia followed by two hours of reoxygenation to induce sI/R injury *in vitro.* The cells were incubated with empagliflozin (EMPA, 10 µM) for 12 h before sI/R injury. **A**, **B** ELISAs were used to analyze the kinase activities of DNA-PKcs and Met in HCAECs following sI/R injury or empagliflozin treatment. **C**–**G** Western blotting was used to determine DNA-PKcs, Met and p-Fis1 protein levels in HCAECs following sI/R injury or empagliflozin treatment. siRNAs were transfected into HCAECs to knock down *DNA-PKcs* oe *Met*. **H**–**J** MitoTracker^™^ was used to detect changes in mitochondrial dynamics. The number cardiomyocyte with fragmented mitochondria as well as the average length of mitochondria were recorded. **K**–**M** To induce oxidative stress, HCAECs were treated with 0.3 mM hydrogen peroxide for six hours to activate DNA-PKcs. NAC (10 mM) was added to the medium in the presence of hydrogen peroxide to reduce oxidative stress-induced DNA-PKcs activation. Then, DNA-PKcs activity was determined with an ELISA. Data are shown as mean ± SEM, n = 10 mice per group or ten independent cell isolations per group. *p < 0.05

Previous studies have identified Met tyrosine kinase [35] and DNA-PKcs[37] as potential upstream triggers of Fis1 phosphorylation; thus, we focused on these two pathways to understand how empagliflozin inhibits Fis1 phosphorylation. Enzyme-linked immunosorbent assays (ELISAs) demonstrated that DNA-PKcs activity was significantly elevated in HCAECs subjected to sI/R injury, while Met activity was not altered (Fig. 6A, B). Additionally, Western blotting illustrated that sI/R injury induced the phosphorylation of DNA-PKcs, but did not alter the expression of Met in HCAECs (Fig. 6C–E). As expected, empagliflozin treatment had no effect on Met activity (Fig. 6 A-B) or expression (Fig. 6C–E), but obviously inhibited DNA-PKcs activity (Fig. 6A, B) and phosphorylation (Fig. 6C–E) in sI/R-treated HCAECs.

**Table 1:** cTnI, CK-MB, LDH, and average blood sugar levels in mouse after I/R injury, n=6 mice per group.

Parameters	Sham	I/R	I/R+EMPA
TnI (ng/ml)	1.07±0.21	16.32±4.27*	8.35±2.46^#^
CK-MB (U/L)	76.2±8.4	215.6±72.6*	103.1±17.7^#^
LDH (U/L)	238.3±42.1	1367.2±174.6*	795.4±86.3^#^
Average blood sugar (mmol/L)	11.2 ± 0.3	17.6 ± 0.4*	13.3 ± 0.4^#^

*p<0.05 vs. sham group,

#p<0.05 vs. I/R group

We then performed loss-of-function assays by using lentiviruses to knock down *DNA-PKcs* or *Met* in HCAECs. Knocking down *DNA-PKcs* suppressed Fis1 phosphorylation in HCAECs subjected to sI/R injury, while knocking down *Met* did not have this effect (Fig. 6F, G). Similarly, sI/R injury-induced mitochondrial fission could be attenuated by empagliflozin treatment or *DNA-PKcs* knockdown, but not by *Met* knockdown in HCAECs (Fig. 6H–J). These data confirmed that DNA-PKcs phosphorylates Fis1 during I/R injury.

Several factors are thought to activate DNA-PKcs, including oxidative stress and DNA damage [50]. Considering that empagliflozin has anti-oxidative properties, we assessed whether empagliflozin might inhibit DNA-PKcs by neutralizing oxidative stress. We added supraphysiological hydrogen peroxide concentrations to the media of empagliflozin-treated sI/R-injured HCAECs. As a negative control group, we treated sI/R-injured HCAECs with the anti-oxidant N-acetylcysteine (NAC). As shown in Fig. 6K, either empagliflozin or NAC treatment could attenuate sI/R injury-induced DNA-PKcs activation; however, exogenous hydrogen peroxide abrogated the ability of empagliflozin to inactivate DNA-PKcs. Likewise, either empagliflozin or NAC supplementation could attenuate sI/R injury-induced mitochondrial fission (Fig. 6J) and Fis1 phosphorylation in HCAECs, but exogenous hydrogen peroxide nullified this effect of empagliflozin (Fig. 6L and M). These results indicated that empagliflozin prevents DNA-PKcs activation by reducing oxidative stress in HCAECs during sI/R injury.

## Discusssion

In the present study, we found that empagliflozin protected against cardiac microvascular I/R injury by reducing mitochondrial fission and inactivating the DNA-PKcs/Fis1 pathway. *In vivo*, empagliflozin administration improved the microvascular structure, reduced luminal stenosis, prevented microthrombus formation and attenuated endothelial hyperpermeability-related inflammatory responses following I/R injury. *In vitro*, empagliflozin enhanced eNOS activity, improved barrier function, maintained the endothelial cytoskeletal integrity and reduced apoptosis in HCAECs subjected to sI/R injury. Mitochondrial dysfunction was found to induce cardiac microvascular endothelial I/R injury. Specifically, an overload of mtROS damaged mitochondrial DNA, thus suppressing the transcription of mitochondria-encoded respiratory complexes and reducing the MMP in HCAECs. However, empagliflozin treatment preserved mitochondrial homeostasis by repressing Fis1-induced mitochondrial fission in these cells. Although empagliflozin also inhibited Drp1 phosphorylation, we found that Fis1 dephosphorylation was the predominant mechanism whereby empagliflozin prevented sI/R injury-induced mitochondrial fission in HCAECs. Indeed, the re-introduction of phosphorylated Fis1 abrogated the protective effects of empagliflozin on mitochondrial homeostasis and endothelial function. Fis1 was primarily phosphorylated by DNA-PKcs in sI/R-injured HCAECs, while empagliflozin could inactivate DNA-PKcs through its anti-oxidative effects. This is the first study to illustrate the mechanism whereby empagliflozin ameliorates cardiac microvascular I/R injury, and suggests that DNA-PKcs/Fis1/mitochondrial fission could be potential targets for the treatment of cardiac microvascular I/R injury.

Empagliflozin has exhibited multiple protective functions in cardiovascular disease studies [51–53]. In myocardia from patients with heart failure with preserved ejection fraction (HFpEF), empagliflozin treatment suppressed the production of inflammatory factors (e.g., ICAM1, vascular cell adhesion molecule 1, TNFα and IL-6) and reversed changes in redox parameters (e.g., H_2_O_2_, 3-nitrotyrosine, glutathione and lipid peroxides) [11]. Through its anti-oxidative effects, empagliflozin attenuated oxidized protein kinase GIα levels, improved myofilament function, reduced cardiomyocyte stiffness and increased the diastolic capacity of the heart [11]. In a murine model of myocardial I/R injury [54], empagliflozin reduced the myocardial infarct size and improved cardiac contractility by stimulating the 5’ adenosine monophosphate-activated protein kinase pathway, independent of its hypoglycemic effects. Empagliflozin was also found to attenuate adverse post-infarcted left ventricular remodeling by elevating the mitophagic flux and enhancing mitochondrial biogenesis [55]. In a mouse model of ventricular fibrillation-induced cardiac arrest [56], empagliflozin treatment improved left ventricular function and increased the survival time, partly by increasing myocardial ketone levels and *β*-hydroxy butyrate dehydrogenase 1 protein expression. Chronic hyperlipidemia was found to augment the cardiomyocyte area and left ventricular thickness, while empagliflozin alleviated this pathological phenotype by inhibiting the myocardial renin angiotensin system, endoplasmic reticular stress and abnormal oxidative reactions [55].

Though numerous studies have examined the effects of empagliflozin on cardiomyocytes, relatively few studies have explored the effects of this drug on endothelial homeostasis and microvascular function, especially under non-diabetic conditions. In heart tissues from HFpEF patients, oxidated eNOS expression was induced, soluble guanylyl cyclase activity was diminished and cyclic guanosine monophosphate levels were reduced relative to control subjects; however, these alterations were undetectable in HFpEF patients receiving empagliflozin [11]. In TNFα-induced endothelial inflammatory injury, empagliflozin inhibited the Na^+^/H^+^ exchanger and reduced cytoplasmic Na^+^ levels, thus suppressing ROS generation [17]. In addition to exerting anti-oxidative effects, empagliflozin was found to activate protein kinase G and voltage-dependent K^+^ channels to dilate the rabbit aorta [57]. Empagliflozin has also been shown to ameliorate other forms of endothelial dysfunction, such as endothelial senescence [58], cellular apoptosis [59], endothelium-leukocyte interactions [60] and endothelial barrier dysfunction [52]. Despite these promising findings, the effects of empagliflozin on cardiac microvascular I/R injury have not been fully understood. Our results suggested that empagliflozin may be an effective tool to attenuate endothelial dysfunction and microvascular injury. Similar to the previous findings, our study demonstrated that empagliflozin can inhibit oxidative stress, inflammation, apoptosis and thrombosis, suggesting that this drug sustains endothelial homeostasis and microvascular integrity through multiple actions.

Mitochondrial damage has been reported as a core pathogenic contributor to endothelial dysfunction under I/R injury [21–23]. Mitochondrial fission is regarded as an early change in mitochondrial morphology that precedes mitochondrial dysfunction. Reperfusion injury was shown to promote Drp1 phosphorylation at Ser616 and rapidly increase the number of fragmented mitochondria, correlating with elevated mtROS production, a reduced MMP and activated mitochondrial apoptosis [41, 61]. On the other hand, inhibiting mitochondrial fission by depleting Drp1 in endothelial cells markedly reduced mtROS generation, restored mitochondrial energy metabolism and promoted cell survival following I/R injury [31]. Moreover, suppressing mitochondrial fission by activating mitophagy or mitochondrial fusion in the endothelium restored eNOS activity [62], improved vascular permeability[63] and normalized endothelium-dependent vascular relaxation [64]. Due to its effects on mitochondrial homeostasis, mitochondrial fission seems to be a good target for the prevention of microvascular I/R injury. Unfortunately, only a few drugs have been reported to inhibit mitochondrial fission and thus protect against microvascular I/R injury – for instance, melatonin [46], angiotensin-converting enzyme inhibitors [65], glucagon-like peptide-1 receptor agonists [66] and green tea (*Camellia sinensis*) [67]. In the present study, we identified empagliflozin as a novel suppressor of mitochondrial fission during I/R-induced endothelial dysfunction. Thus, empagliflozin may be able to attenuate perioperative microvascular injury in patients receiving revascularization treatment.

Empagliflozin has already been widely reported to inhibit mitochondrial fission in various cell types, including adipocytes [68], renal tubular cells [69] and endothelial cells[32]. It is generally accepted that empagliflozin suppresses mitochondrial fission by reducing Drp1 phosphorylation [33, 34]. In the present study, we also found that empagliflozin prevented Drp1 phosphorylation in HCAECs subjected to sI/R injury. After translocating from the cytoplasm to the surface of mitochondria, Drp1 consumes ATP and divides mitochondria into several fragments [49]. Although phosphorylation increases the mobility of Drp1 [70], the binding of cytoplasmic Drp1 to mitochondria requires several receptors, including Mff and Fis1 [71, 72]. The phosphorylation of Fis1 [35] and Mff [36] increases their affinity for cytoplasmic Drp1, and thus is important for inducing mitochondrial fission, regardless of whether Drp1 itself is phosphorylated. In the present study, we found that the phosphorylation of Fis1, but not Mff, was induced by sI/R injury and inhibited by empagliflozin in HCAECs. Transfection of a Fis1 phosphorylation-mimetic mutant, rather than a Drp1 phosphorylation-mimetic mutant, prevented empagliflozin from inhibiting mitochondrial fission in sI/R-injured HCAECs. These data suggested that Fis1 dephosphorylation, rather than Drp1 dephosphorylation, is the primary mechanism whereby empagliflozin inhibits mitochondrial fission in the endothelium following I/R injury. Lastly, we demonstrated that empagliflozin blocked Fis1 phosphorylation by preventing DNA-PKcs activation in sI/R-injured HCAECs; however, exogenous hydrogen peroxide treatment offset this effect. Therefore, through its anti-oxidative properties, empagliflozin prevented DNA-PKcs activation and hindered its kinase activity, ultimately reducing Fis1 phosphorylation.

This study had several limitations. First, it is difficult to evaluate microvascular function *in vivo*, so we only observed structural changes in microvessels following empagliflozin treatment. Second, we were only able to perform molecular investigations *in vitro* due to the fatality of Fis1 or DNA-PKcs transgenic mice.

## Conclusions

Our results demonstrated that empagliflozin can protect against cardiac microvascular I/R injury. Empagliflozin administration prevented oxidative stress-induced DNA-PKcs activation, thus suppressing Fis1 phosphorylation and mitochondrial fission in sI/R-injured HCAECs. By inhibiting mitochondrial fission, empagliflozin sustained mitochondrial homeostasis, thereby improving endothelial function and protecting the microcirculation during cardiac I/R injury. Based on our results, empagliflozin should be considered as a potential microvascular protective drug that inhibits the DNA-PKcs/Fis1/mitochondrial fission pathway.

## Data Availability

All data generated or analyzed during this study are included in this published article.
